# Concurrent Optical- and Magnetic-Stimulation-Induced Changes on Wound Healing Parameters, Analyzed by Hyperspectral Imaging: An Exploratory Case Series

**DOI:** 10.3390/bioengineering10070750

**Published:** 2023-06-23

**Authors:** Jürg Traber, Thomas Wild, Jörg Marotz, Martin C. Berli, Alfredo Franco-Obregón

**Affiliations:** 1 Venenklinik Bellevue, Brückenstrasse 9, 8280 Kreuzlingen, Switzerland; 2 Clinic of Plastic, Hand and Aesthetic Surgery Burn Center, BG Clinic Bergmannstrost, 06112 Halle (Saale), Germany; 3 Medical University Halle, Outpatient and Operating Center, Martin-Luther University Halle (Saale), 06112 Halle (Saale), Germany; 4 Institute of Applied Bioscience and Process Management Head of Education Course “Academic Wound Consultant”, University of Applied Science Anhalt, 06366 Koethen, Germany; 5 BG-Klinikum Bergmannstrost, 06112 Halle (Saale), Germany; 6 Department of Surgery, Spital Limmattal, 8952 Schlieren, Switzerland; 7 Department of Surgery, Yong Loo Lin School of Medicine, National University of Singapore, Singapore 119228, Singapore; 8 Institute of Health Technology and Innovation (iHealthtech), National University of Singapore, Singapore 117599, Singapore; 9 Biolonic Currents Electromagnetic Pulsing Systems Laboratory (BICEPS), National University of Singapore, Singapore 117599, Singapore; 10 Competence Center for Applied Biotechnology and Molecular Medicine, University of Zürich, 8057 Zürich, Switzerland

**Keywords:** concurrent optical and magnetic stimulation (COMS), hyperspectral imaging, chronic wounds, tissue oxygenation, modulation inflammation, advanced wound care, hard-to-heal wounds

## Abstract

The effects of concurrent optical and magnetic stimulation (COMS) therapy on wound-healing-related parameters, such as tissue oxygenation and water index, were analyzed by hyperspectral imaging: an exploratory case series. **Background**: Oedema and inadequate perfusion have been identified as key factors in delayed wound healing and have been linked to reduced mitochondrial respiration. Targeting mitochondrial dysfunction is a promising approach in the treatment of therapy refractory wounds. This sub-study aimed to investigate the effects of concurrent optical and magnetic stimulation (COMS) on oedema and perfusion through measuring tissue oxygenation and water index, using hyperspectral imaging. **Patients and methods**: In a multi-center, prospective, comparative clinical trial, eleven patients with chronic leg and foot ulcers were treated with COMS additively to Standard of Care (SOC). Hyperspectral images were collected during patient visits before and after treatment to assess short- and long-term hemodynamic and immunomodulatory effects through changes in tissue oxygenation and water index. **Results**: The average time for wound onset in the eleven patients analyzed was 183 days, with 64% of them being considered unresponsive to SOC. At week 12, the rate of near-complete and complete wound closure was 64% and 45%, respectively. COMS therapy with SOC resulted in an increased short-term tissue oxygenation over the 8-week treatment phase, with oxygen levels decreasing in-between patient visits. The study further found a decrease in tissue water content after the therapy, with a general accumulation of water levels in-between patient visits. This study’s long-term analysis was hindered by the lack of absolute values in hyperspectral imaging and the dynamic nature of patient parameters during visits, resulting in high interpatient and intervisit variability. **Conclusions**: This study showed that COMS therapy as an adjunct to SOC had a positive short-term effect on inflammation and tissue oxygenation in chronic wounds of various etiologies. These results further supported the body of evidence for safety and effectiveness of COMS therapy as a treatment option, especially for stagnant wounds that tended to stay in the inflammatory phase and required efficient phase transition towards healing.

## 1. Introduction

Wound healing is a well-tuned biological process, which is achieved via consecutive and overlapping phases, including hemostasis, inflammatory-related events, cell proliferation, and tissue remodeling [1,2]. The orchestration of these interconnected molecular cascades requires sufficient mitochondrial respiration and intracellular energy to enable these processes [3,4].

Oedema and inadequate perfusion are the main reasons for delayed wound repair and have been linked to reduced mitochondrial respiration and intracellular ATP production, which are essential for providing sufficient intracellular energy for tissue regeneration [5,6,7]. Therefore, targeting mitochondrial dysfunction and inflammation are promising approaches in the treatment of therapy refractory wounds [6,8,9]. Thus, assessing changes in wound-healing-related parameters associated with tissue perfusion and oedema through hyperspectral imaging, such as tissue oxygenation and water index, are important to understand the effectiveness of novel treatment modalities [10,11].

A recent publication has demonstrated the effectiveness of concurrent optical and magnetic stimulation (COMS) in the promotion of wound healing in hard-to-heal wounds of various etiologies [12]. However, the underlying mechanism of action has not yet been sufficiently clarified. The aim of this sub study was to investigate the potential hemodynamic effects of COMS and its modulation of inflammation, which are essential to set the base for efficient phase transition towards wound healing, with hyperspectral imaging.

### 1.1. Resolving Photomagnetic Transduction

A current challenge in the field of bioelectromagnetism is coalescing what is known about magnetism and photomodulation into one unifying cellular response. According to one form of the radical pair mechanism (RPM) of magnetoreception, the photoexcitation of a semiquinone flavin enzyme in the presence of molecular oxygen leads to the production of a spin correlated electron radical pair between the reduced flavoenzyme (FADH•) and the resulting superoxide radical (O2•−) that is favored by the presence of an external weak magnetic field [13] and serves to modulate mitochondrial respiration via the electron transport system containing cytochrome C [14].

Another version of the RPM implicates a crytochrome moiety to form a radical pair with FADH• that then activates a downstream molecular cellular transducer in the presence of a magnetic field [15], such as an ion channel [16]. Crytochromes also have been shown to underlie photomechanical transduction [17] and circadian cell cycle regulation in skeletal muscle cells supported by calcium entry [18].

Notably, photomodulation has been proposed to invoke the participation of transient receptor potential (TRP) channels [19]. One form of photomodulation specifically implicates the activation of TRP channels via their coupling to melanopsin [20,21] that plays a role in thermoregulation, circadian cell cycle regulation, pigmentation development, and Vitamin D synthesis [22].

Broadly speaking, TRP channels can be envisioned as integrators of diverse forms of biophysical stimuli (mechanical force, light, temperature, pH, etc.) [23,24], which then transduce their biophysical activation into mitochondrial cellular responses [25,26]. Reactive oxygen species (ROS) also stimulate TRPC1-mediated calcium entry [27], which, in turn, stimulate mitochondrial respiration [28,29], reinforcing the response. Therefore, by impinging on TRPC1, magnetic and optical stimulation are capable of synergizing their effects to promote tissue regeneration.

### 1.2. Mechanism of Action Concurrent Optical and Magnetic Stimulation (COMS)

The concurrent application of optical and magnetic stimulation is intended to complement each other and has significant positive effects on almost all phases of tissue repair. While magnetic stimulation (MS) is associated with immunomodulatory action [30], stimulation of tissue perfusion [31], enhanced proliferation, differentiation, and survival [28,32], optical stimulation (OS) induces cell proliferation [33] and connective tissue remodeling [34]. The effects of COMS on wound-related parameters such as tissue perfusion and oedema are still insufficiently investigated.

**Figure 1:**
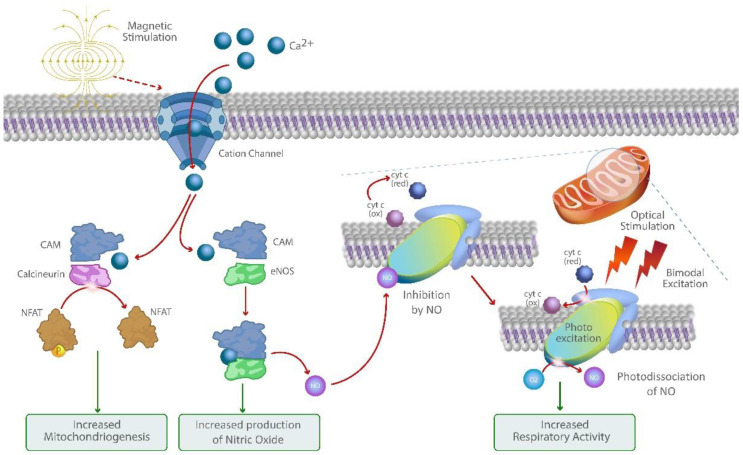
Graphic illustration of the molecular mechanism of action of COMS. Bimodal photo biomodulation of cytochrome C oxidase amplifies Ca^2+^ and NO-mediated signaling pathways activated through magnetic stimulation of cation channels and recovers respiratory activity and ATP production.

Although the mechanism of action of optical stimulation is well described in the literature, the underlying mechanisms of action on a molecular level of magnetic stimulation remained largely elusive [35,36]. Recent publications have demonstrated the importance of mechanosensitive (MS) ion channels in regulating processes such as skin cell proliferation, differentiation, and barrier formation and linked their dysfunction to dermatological disorders [37,38,39]. Magnetic stimulation has been shown to activate mechanosensitive TRPC1 channels, whereby the resulting ion-channel-mediated calcium fluxes stimulate mitochondrial respiration and associated mitochondriogenesis through the activation of the Ca^2+^/CalModulin(CaM)/NFAT/PGC-1α pathway (Figure 1) [28,40]. Increased intracellular Ca^2+^ promotes interactions between calmodulin and eNOS and leads to enhanced eNOS activity and nitric oxide (NO) production, a potent mediator for vasodilation and tissue perfusion [41].

The concurrent bimodal red and near-infrared photo biomodulation can further increase mitochondrial respiration and intracellular ATP production through the activation of the cytochrome C oxidase and mitigating its downregulation by increased levels of intracellular NO through its photo dissociation [42,43]. Through this synergistic mechanism, COMS may offer a therapeutic option for amplifying Ca^2+^ and NO-mediated processes such as cell proliferation, migration, vasodilation, and angiogenesis while helping to shift the cytokine profile towards resolution of inflammation. Therefore, COMS may offer a promising therapeutic approach to enable the transition towards the healing of therapy refractory wounds that tend to remain in the inflammatory phase.

## 2. Patients, Materials, and Methods

### 2.1. Parameters for Concurrent Optical and Magnetic Stimulation 

Pulsed modulated magnetic fields in the extremely low frequency (ELF) range were produced with a coil. The signal was emitted in an asymmetrical trapezoidal shape with a 20 Hz frequency, with an increasing peak field strength up to 1.6 mT (16 Gauss) over time at the treatment location, for a duration of 16 min per session. The optical stimulation component was designed to emit light using two types of LEDs in the wavelengths of 660 nm (red) and 830 nm (near-infrared (NIR)). The optical signal was pulsed at 1 kHz with a maximum pulse width of 0.3 ms. A pulse peak power of 25 mW/cm^2^ was emitted at an average power output of 5 mW/cm^2^ at the treatment area.

### 2.2. Hyperspectral Imaging

Hyperspectral imaging (HSI) has established itself as a novel diagnostic tool to assess the effect of novel treatment modalities on wound-healing-related parameters such as oedema and tissue oxygenation, delivering real-time tissue characterization and treatment guidance.

HSI is based on optical remission spectroscopy in the visible and NIR spectral range. In this range, the penetration depth in the skin is between a tenth of a millimeter (visible) and 4–5 mm (NIR). In this spectral range, the main components of the skin, especially hemoglobin but also other chromophores, exhibit a significant dependency of their scattering and absorption properties on the wavelength. Due to this physical interaction, the remitted light contains information about these components, especially about the perfusion represented by reduced hemoglobin and oxygenated hemoglobin (HbO_2_) [44]. All measurements were performed with the HSI camera TIVITA^®^ Tissue (Diaspective Vision GmbH, Germany). The HSI camera is a compact measuring system certified for clinical use that includes an illumination together with a reception unit. Remission spectra were recorded in the spectral range from 500 to 1000 nm with a resolution of 5 nm, a measurement area of approximately 20–30 cm^2^, and standard image size of 640 × 480 pixels. The recording required approximately five seconds. The camera system allowed for quick and uncomplicated measurements without the need for special measurement conditions, except for the avoidance of external light illumination.

### 2.3. HSI Processing

The information contained in the remission spectra was extracted using a concrete model of the measured tissue volume by approximately solving the “inverse problem” (calculation of model parameters from the spectral data). However, as the penetration depth depended on the wavelength (λ), the remission spectrum was heterogeneous, meaning that the measured volume was different and captured different tissue layers depending on λ. Due to this fact and the large number of parameters in the realistic model, the inverse problem could be solved practically only under simplifying assumptions. To overcome the restrictions of the customarily used simple homogenous one-layer model, TIVITA used parameters based on a more realistic six-layer model to allow for a more differentiated analysis of the perfusion. We developed a more realistic model for data processing of the remission spectra, allowing to calculate depth profiles of the perfusion (three-dimensional physiological perfusion imaging) [45]. Using this processing method, the information content of the spectra was evaluated more extensively.

### 2.4. Patients and Data Collection

HSI measurements were collected in 11 patients recruited during a multi-center, prospective, comparative clinical study at a single center in Switzerland (Venenklinik Bellvue AG). The study was approved by the Ethics Committee Ostschweiz, Swissmedic reference number 2017-MD-0008 (ClinicalTrials.gov Identifier: NCT03112395) according to the requirements of the International Conference on Harmonization Good Clinical Practices (ICH GCP) guidelines based on the ethical principles of the Declaration of Helsinki. The aim of this sub study was to assess and visualize the hemodynamic and immunomodulatory effects of COMS treatment in therapy refractory ulcers of different etiology.

**Figure 2:**
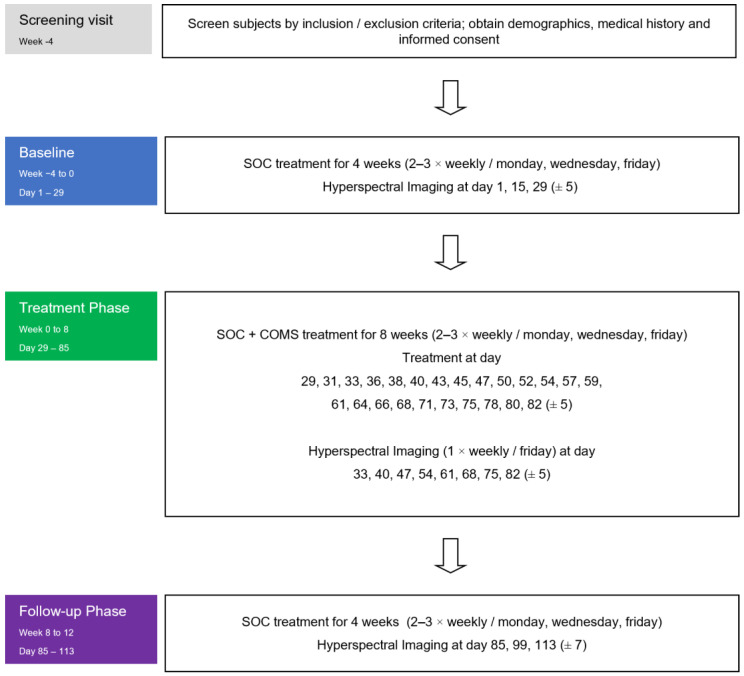
Study flowchart: Hyperspectral images were collected in all three phases of the study at the patient visits (always before and after the performed treatments).

Hyperspectral images were collected as a surrogate endpoint to analyze the effects of COMS treatment on wound-healing-related parameters such as tissue oxygenation and tissue water index (Figure 2). HSI measurements were collected during baseline, treatment, and follow-up phase. Measurements were performed during patient visits before and after COMS treatment as an adjunctive to Standard of Care (SOC). During the study period, the standard of care (SOC) for wound treatment was based on the recommendations of the Association for Wound Care (SAWC) but was individualized as deemed necessary by the treating physicians. The SOC comprised wound cleansing, debridement, exudate management, maintaining an appropriate level of moisture for the wound, and addressing infection and inflammation. The use of any other advanced wound care products was not permitted. Additionally, addressing the vascular cause of the wound, such as using compression therapy, was considered SOC and was continuously addressed throughout the study.

### 2.5. Measurement of Wound States with Hyperspectral Imaging

The state of a wound can be characterized by factors such as its size, the proportion of different types of wound tissue (such as necrosis, fibrin, minimally perfused critical tissue, granulation, and epithelization), as well as the perfusion and water content in the deeper wound area. Perfusion can be quantified by measuring deep blood flow, including the volume and oxygenation of hemoglobin. A sufficient deep perfusion and oxygen supply, combined with angiogenesis, can result in improved overall wound perfusion, serving as a crucial indicator of the wound’s macroscopic state. Enhanced water content may characterize ongoing inflammation, and the removal of this water helps to shift the cytokine profile towards the resolution of inflammation.

For the assessment of the wound healing process through the study period of 12 weeks, the quantified change in the state in the course of time as a reaction to the treatment is an expressive measure. With his, a simple and contact-free method was available, enabling a detailed and quantitative description of the wound state [44].

Remission spectroscopy in the spectral range from approx. 500 to 1000 nm [10,46,47] enabled

- the determination of the wound size by imaging;
- the determination of the mentioned different tissue types using specific spectral features (also with quantitative specification of this types);
- the determination of the perfusion (volume fraction and oxygenation of the hemoglobin) in the wound area (superficial for critical tissue and granulation and deep for the wound ground) by using the specific absorption of hemoglobin;
- the general perfusion quality of the wound environment, describing systemic factors responsible for wound development;
- the determination of the relative water content.

Considering the altered structure of wounds in comparison to normal skin and to obtain comparable parameters, the evaluation has been focused on the perfusion parameter in the depths of the wound.

Based on this parameter, on the macroscopic level, a significant quantitative description of the wound state and the change in the state in the course of time was provided as an objective basis for comparability of different wounds and the evaluation of specific wound treatment methods in studies.

**Figure 3:**
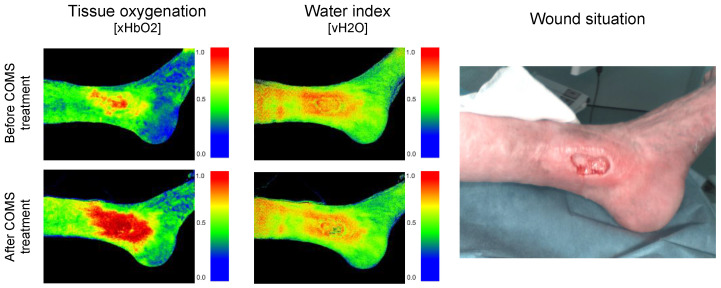
Hyperspectral Imaging measuring changes in deep blood flow, including the volume and oxygenation of hemoglobin as well as water content before and after COMS therapy in the wound area.

The combination of the COMS method affecting the wound on a molecular level with the objective macroscopic wound description by HSI enabled the differentiation between effects induced by COMS and systemic or environmental variations in the perfusion quality (Figure 3).

## 3. Results

### 3.1. Patient Demographics and Wound Size Changes in Enrolled Patients

In this sub study, 11 individuals with chronic leg and foot ulcers were examined using HIS (Table 1). Out of the 11 ulcers studied, 8 were identified as venous leg ulcers (VLU), and the remaining 3 were classified as mixed leg ulcers (MLU). The size of the wounds varied from 2.1 to 29.6 cm^2^, with an average of 11.7 cm^2^ and a median of 8.7 cm^2^. The average wound onset was 183 days, and the median was 92 days, indicating that a majority of patients were classified as therapy refractory based on EWMA and FDA guidelines [48,49,50]. Both genders were represented by five females and six males.

Wound size was evaluated at two different times to assess the patients’ responsiveness to the standard of care (SOC) and the combination of COMS treatment. The first assessment was performed at the end of SOC baseline (day 29) and after the treatment follow-up phase at week 12 (day 113). On day 29, 36% of the patients saw a reduction in wound size of more than 30% solely from improved compliance with SOC regulations and were classified as SOC responders. On the other hand, 64% of patients were considered therapy refractory and non-responders to SOC, showing little-to-no change in wound size (less than 30% reduction) during the 4-week (29 days) SOC baseline period.

Additionally, the rate of near-complete and complete wound closure, defined as a total wound area reduction of 90% and 100%, respectively, were assessed. At day 113, 64% of patients reached a near-complete wound closure (area reduction of > 90%), and 45% of patients had fully healed (Table 1). Overall, by week 12 after COMS initiation, 82% of patients had seen a reduction of more than 50% in their respective wound size. However, two patients (patient four and seven) did not have a positive response to the treatment, showing a less than 50% reduction in wound area. These two were, therefore, categorized as COMS non-responders. Patient seven was especially unresponsive, as the wound size increased over the 12-week study period.

### 3.2. Long-Term Changes in Tissue Oxygenation over Study Period

To evaluate wound-healing-related parameters during the healing progress of wounds, HSI was used to measure the changes in deep tissue oxygenation within the wound and its environment during two time periods, namely, the SOC baseline phase from day 1 to day 29 and the follow-up phase from day 81 to day 113. This approach was used to try to understand the long-term impact of COMS therapy on deep tissue oxygen perfusion after the 8-week treatment phase. The average values from the baseline and follow-up phase did not provide clear patterns in changes in deep tissue oxygenation (xHbO2) and did not correlate with wound area reductions measured over time. 

### 3.3. Short-Term Changes in Tissue Oxygenation before and after COMS Therapy

**Figure 4:**
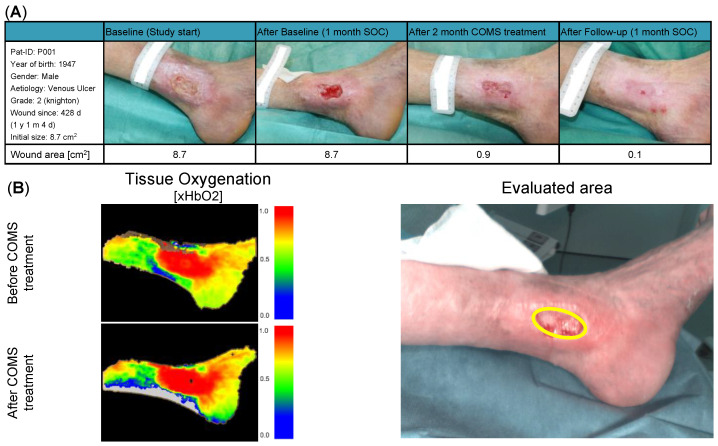
(**A**) Patient description and demographic: Patient P001 presented with a grade two venous leg ulcer according to Knighton, which had been present on the medial left ankle for 428 days at the start of the study. During the baseline period, the patient did not show a significant reduction in the wound area, indicating non-responsiveness to standard of care (SOC) treatment. During COMS treatment, the wound area was markedly reduced to 0.9 cm^2^, with the wound achieving a near-complete closure (0.1 cm^2^) at day 113. (**B**) Short-term changes in tissue perfusion: Case example patient 001. Visualization of changes in tissue oxygenation (xHbO2) before and after COMS therapy, measuring deep blood flow and oxygenation of hemoglobin.

Given the high interpatient and inter-visit variability present, as reported in the last section, delta values in-between treatment visits were analyzed. Therefore, the average change in short-term tissue oxygenation, namely, a period of approximately 30 min after SOC+COMS application, as well as the delta in-between two treatment visits, namely, a time period of 2–3 days in-between dressing changes, were assessed during the 8-week SOC+COMS treatment period (day 31 to day 84). This enabled us to evaluate the short-time impact of COMS therapy on tissue oxygenation and provided a meaningful conclusion about its underlying relevance for promoting wound healing (Figure 4).

### 3.4. Average Short-Term Changes in Tissue Oxygenation over COMS Treatment Period

**Figure 5:**
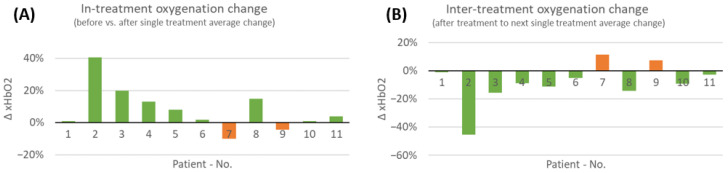
(**A**) Average changes in short-term tissue oxygenation analyzed during the 8-week COMS treatment period. The in-treatment change was analyzed before and after SOC+COMS treatment. (**B**) Further the inter-treatment changes in tissue oxygenation in-between dressing changes were analyzed. Average changes in short-term tissue oxygenation at inter-treatment visits, analyzed in-between dressing changes every 2–3 days during COMS treatment period.

Considering all performed measurements before and after COMS therapy, an overall increase in tissue oxygenation was found in 9 out of 11 patients (Figure 5). However, it should be noted that patients seven and nine had an overall negative reaction, with a short-term reduction in oxygenation over all performed measurements of the treatment period. Patient seven seemed to have a different reaction to the therapy compared to other patients.

While oxygenation levels acutely increased in most cases with COMS treatment, oxygenation levels in-between therapy sessions generally decreased. This was observed in all patients, except for the same two patients that did not show an increase in short-term oxygenation directly after COMS therapy, indicating that these wounds responded differently to the therapy compared to others.

### 3.5. Changes in Tissue Water Content Changes over Study Period

Furthermore, changes between the SOC baseline phase (day 1 to day 29) and COMS follow-up phase day (81 to day 113) in tissue water index within the wound were analyzed and compared. Again, the findings were consistent with those of oxygenation, showing no patterns in changes in tissue water index over the course of COMS therapy and not correlating with the wound area reduction experienced by patients.

### 3.6. Short-Term Changes in Water Content before and after COMS Therapy

**Figure 6:**
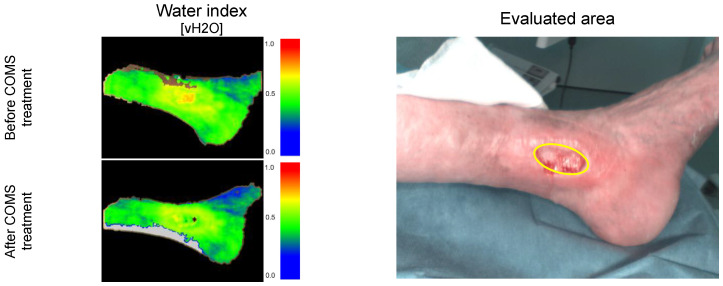
Short-term changes in tissue water oxygenation: Case example patient 001. Visualization of water deposition (vH2O) before and after COMS therapy, measuring deep lymphatic flow and drainage.

Given the high variability present in long-term evaluations, the study team again focused on analyzing the short-time delta for tissue water content before and after COMS therapy sessions, similar to the approach used for measuring short-term changes in tissue oxygenation (Figure 6).

### 3.7. Average Short-Term Changes in Water Content over COMS Treatment Period

**Figure 7:**
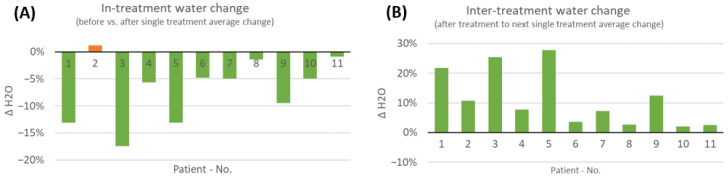
(**A**) Average changes in short-term water tissue index analyzed during the 8-week COMS treatment period. The changes were analyzed before and after SOC+COMS treatment. (**B**) Average changes in short-term water tissue index at inter-treatment visits, analyzed in-between dressing changes every 2–3 days during COMS treatment period.

The results showed that there was a short-term effect in reducing tissue water within the wound and its environment in 10 out of 11 patients on average (Figure 7). This effect was accompanied by elevated levels of tissue secretion during the treatment sessions noted by the care givers. Only the wound of patient two had seen an increase in water content after the treatment. Nevertheless, patient two (a SOC responder) was already healed by 79% at therapy start and, subsequently, completely healed during the study period. Interestingly, when analyzing the changes in tissue water content in-between treatment visits, water levels accumulated in the days between treatments for all patients.

### 3.8. Case Reports COMS Non-Responder Patient 004 and 007

**Figure 8:**
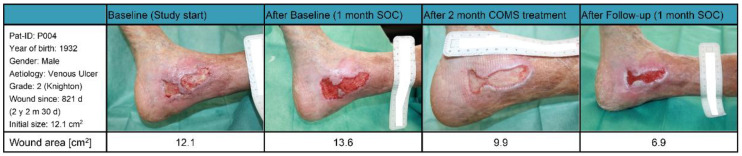
Case report patient 004.

Patient 004 (Figure 8): The male patient, who was born in 1932 with a BMI of 20.7 kg/m^2^, had a complex medical history that included dilatative arteriopathy for almost a decade, chronic obstructive pneumopathy for eight years, and hypertensive coronary disease for four years. At the beginning of the study, the patient was presented with a venous leg ulcer (Knighton grade two) on his right ankle, which had been present for over two years (821 days) and had a wound area of 12.1 cm^2^. After receiving 29 days of baseline standard of care (SOC) treatment, he was classified as a SOC non-responder, with the wound area increasing to 13.6 cm^2^, prior to starting COMS treatment.

The COMS treatments provided short-term decreases in tissue water index and increases in tissue oxygenation. The wound tissue composition improved, which correlated with a decrease in wound size of 43% by week 12. It could be reasoned that the wound would have fully closed if the treatment was continued for a longer period of time. In this stagnant and therapy-resistant wound, COMS therapy was finally able to support the resolution of inflammation and setting the foundation for the healing.

**Figure 9:**
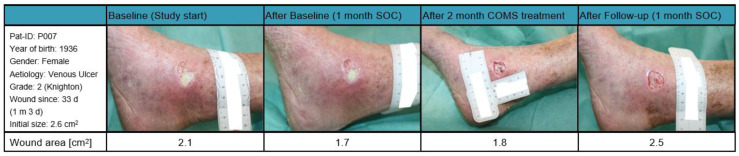
Case report patient 007.

Patient 007 (Figure 9): A female patient born in 1936 with a BMI of 19.56 kg/m^2^, presented with a grade two ulcus cruris venosum on the medial right ankle, according to the classification by Knighton with a wound area of 2.1 cm^2^ at study start. The wound had been present for 33 days prior to enrollment in the study and did not show significant reduction in wound size during the first month of SOC treatment and was, therefore, categorized as a SOC non-responder. The patient reported constant pain and felt pressure on the wound while sleeping.

During the COMS treatment, the patient exhibited an atypical decrease in tissue oxygenation followed with a reduction in tissue water index. This response may have been due to her comorbidities, such as inadequate vascular prefusion or blockages that could have negatively affected the response to the therapy. Overall, the wound area gained in size over the study period of 12 weeks. It is to be noted that there was a shift in tissue composition from mainly fibrotic to granulation during the last weeks of COMS treatment, accompanied with a decrease in wound size prior to the follow-up phase.

## 4. Discussion

The process of wound healing involves a complex interplay of cellular interactions, proteases, growth factors, and extracellular matrix components. Adequate oxygen supply is crucial for these wound healing processes, providing energy for cellular activities such as fibroblast proliferation, collagen deposition, and angiogenesis [3,4]. Chronic wounds, in particular, are prone to hypoxia due to high metabolic demands, making adequate tissue perfusion and oxygen supply important parameters essential to set the base for efficient phase transition towards healing trajectory [5,6,7]. As expected, among the targeted patient population suffering from chronic ulcers of a vascular origin, accompanying pathological microcirculation issues were found in most patients’ documented medical history (73% of the study population). Additionally, the modulation of inflammation was crucial, as chronic inflammation could prolong the healing process and lead to the development of therapy-refractory wounds [6,8,9]. Therefore, controlling inflammation was essential to prevent chronic wounds from becoming unresponsive to therapy. Oedema and the accumulation of water in the wound tissue is often associated with inflammation, making the activation of lymphatic drainage a valuable option to modulate inflammation by removing excess fluids and reducing oedema.

This study found interesting data when it came to the short-term changes in tissue oxygenation and water content. The majority of patients showed increased short-term tissue oxygenation after receiving COMS therapy as an adjunct to SOC. This increase is believed to be due to the Ca^2+^-mediated increases in nitric oxide levels [41], leading to improved blood flow and tissue perfusion through the vasodilation of blood vessels and promotion of angiogenesis. The study also observed that oxygen levels often decreased over time in-between therapy sessions. This decrease is hypothesized to result from the modulation of cytochrome C oxidase activity [42,43], leading to the increased consumption of oxygen through mitochondrial respiration and intracellular ATP production. As a result, the tissue’s oxygen may have been used up more quickly, leading to a decrease in oxygenation levels.

In addition, the study found that the majority of patients showed a short-term decrease in tissue water content after receiving COMS therapy as an adjunct to SOC. The decrease in tissue water content is believed to be caused by the activation of lymphatic drainage through increased levels of nitric oxide. The increase in lymphatic flow removes waste and excess fluid from the wound site and may have helped in promoting the healing process and a faster resolution of the inflammation. The analysis of changes in tissue water content in-between treatment visits showed a general accumulation of water levels during dressing changes, both in the wound itself and in its surrounding environment. Although this accumulation did not appear to have an immediate impact on wound healing, it is still an important factor to consider in the overall wound progression. It is to be noted that it is not possible to remove any water from the body in the absence of oedema. Consequently, certain patients may not experience a reduction in water content despite being healed, as observed in patient two in this study. The results suggested that COMS therapy was successful in modulating inflammation and delaying potential negative effects of oedema on healing. This indicated that the COMS therapy may not have only improved tissue perfusion and reduced tissue water content but also played a role in preventing unwanted accumulation of fluid that could negatively impact wound healing.

When trying to compare long-term data, it was seen that values from hyperspectral imaging were no absolute values, and patient parameters tended to change in-between patient visits, making obtaining accurate and consistent data challenging. Therefore, comparisons between baseline and follow-up data did not show any patterns whatsoever. Despite the limitations on comparing long-term data, it is important to note that this was the first investigation analyzing wound-healing-associated parameters over a prolonged period to the authors knowledge, demonstrating the limitations of utilizing hyperspectral imaging for long-term evaluations. Though, further analysis that focused on the short-term effect, where consistent effects were demonstrated over longer periods of time using hyperspectral imaging, provided interesting data. It allowed for analysis of changes in tissue perfusion and lymphatic flow induced by COMS therapy, shedding light on the potential mechanisms of action of COMS therapy and providing insights for future clinical investigations.

In the patient population the average onset time for wounds was 183 days, with 64% of them being considered unresponsive to standard of care treatment. However, wound closure rates were noteworthy, with 45% of patients fully healed by week 12 and 64% demonstrating a near-complete closure. Overall, 82% of patients profited from COMS therapy with a decrease of more than 50% in wound size by week 12. Nevertheless, in this sub study two patients (4 and 7) were found to have no significant treatment reaction, associated with a disproportional reduction in wound size (less than 50% reduction). The reason for this unresponsiveness is speculated to have been due to underlying comorbidities such as inadequate vascularity, which prevented COMS therapy from increasing tissue perfusion and promoting angiogenesis. For patient seven, these findings correlated with the observed unresponsiveness to the improved perfusion of COMS. For patient four, with the most extended wound onset, being unresponsive to various treatment modalities for over 821 days prior to recruitment in the study, the COMS therapy was finally able to support the resolution of inflammation and setting the foundation for the healing.

Future studies should focus on identifying the underlying factors that contribute to the varying responses to COMS therapy to develop more personalized treatment plans and to improve outcomes for patients with long-standing chronic leg and foot ulcers. Additionally, it would be beneficial to investigate the long-term outcomes of patients who did respond well to the treatment, to determine other important factors such as the durability of the wound closure and the risk of wound recurrence.

## 5. Conclusions

In conclusion, while uncovering limitations in comparing wound-healing-related parameters assessed through hyperspectral images over extended periods of time, this study demonstrated its use to investigate short-term changes in wound-healing-related parameters over longer treatment durations. This exploratory sub study demonstrated positive short-term effects of COMS therapy as an adjunctive treatment to standards of care on modulation of inflammation and increase in tissue perfusion in chronic wounds of various etiologies. These are important observations that help to shed light on the underlying physiological conditions required to set the foundation for efficient phase transition towards the healing of wounds that tend to stay in the inflammatory phase. In addition, the results of this study add to a growing body of evidence, confirming the safety and efficacy of COMS therapy as a new treatment option for patients with chronic wounds, particularly those who did not respond to standard of care (SOC) treatment.

## Figures and Tables

**Table 1:** Characteristics of the patient sample population, including time between wound onset and screening by patient and change in wound area between baseline (day 29) and follow-up (day 113).

Patient ID	Indication	Wound Onset (Days)	Wound Size at Baseline (cm²)	Area Reduction Day 29	Area Reduction Day 113
001	VLU	428	8.7	0%	**99%**
002	VLU	43	2.4	79%	**100%**
003	VLU	94	5.5	22%	**53%**
004	VLU	821	12.1	−12%	**43%**
005	VLU	33	3.7	49%	**100%**
006	MLU	60	29.6	6%	**100%**
007	VLU	33	2.1	19%	**−19%**
008	MLU	214	11.2	19%	**100%**
009	MLU	67	21.6	17%	**72%**
010	VLU	128	26.8	68%	**93%**
011	VLU	92	5.4	91%	**100%**

## Data Availability

The data presented in this study are available in the article.
